# Green Tea Prevents NAFLD by Modulation of miR-34a and miR-194 Expression in a High-Fat Diet Mouse Model

**DOI:** 10.1155/2019/4168380

**Published:** 2019-12-04

**Authors:** L. F. Torres, B. Cogliati, R. Otton

**Affiliations:** ^1^Interdisciplinary Post-Graduate Programme in Health Sciences, Cruzeiro do Sul University, São Paulo, Brazil; ^2^Department of Pathology, School of Veterinary Medicine and Animal Science, University of São Paulo, São Paulo, Brazil

## Abstract

**Background/Aims:**

Nonalcoholic fatty liver disease (NAFLD) is considered the hepatic manifestation of metabolic syndrome. It is currently the most common chronic liver disease with complex pathogenesis and challenging treatment. Here, we investigated the hepatoprotective role of green tea (GT) and determined the involvement of miRNAs and its mechanism of action.

**Methods:**

Male C57Bl/6 mice were fed with a high-fat diet for 4 weeks. After this period, the animals received gavage with GT (500 mg/kg body weight) over 12 weeks (5 days/week). HepG2 cell lines were transfected with miR-34a or miR-194 mimetics and inhibitors to validate the *in vivo* results or were treated with TNF-*α* to evaluate miRNA regulation.

**Results:**

GT supplementation protects against NAFLD development by altering lipid metabolism, increasing gene expression involved in triglycerides and fatty acid catabolism, and decreasing uptake and lipid accumulation. This phenotype was accompanied by miR-34a downregulation and an increase in their mRNA targets *Sirt1*, *Pparα*, and *Insig2*. GT upregulated hepatic miR-194 by inhibiting TNF-*α* action leading to a decrease in miR-194 target genes *Hmgcs/Apoa5*.

**Conclusion:**

Our study identified for the first time that the beneficial effects of GT in the liver can be due to the modulation of miRNAs, opening new perspectives for the treatment of NAFLD focusing on epigenetic regulation of miR-34a and miR-194 as green tea targets.

## 1. Introduction

Nonalcoholic fatty liver disease (NAFLD) is currently the most common chronic liver disease in Western countries, affecting about 30% of the worldwide population [1–3]. The NAFLD is considered the hepatic manifestation of metabolic syndrome and it is highly related to obese patients [4]. The mechanisms underlying the development and progression of NAFLD are displayed in a multiple hit model that involves insulin resistance (IR), nutritional factors overload, gut microbiota dysfunction, endoplasmic reticulum (ER) stress, inflammatory liver environment (cytokine release), and genetic and epigenetic factors [5–7].

Green tea (GT) is one of the most consumed beverages in the world and its high content of polyphenols guarantees health beneficial effects. Among the main biological effects attributed to GT polyphenols, we can highlight the antioxidant, anti-inflammatory, antitumor, antidiabetic, antiobesity, and hepatoprotective activities [8, 9]. Several studies have related the GT consumption to the improvement and prevention of NAFLD [10–13]. The administration of EGCG, the main polyphenol in GT, improves liver function and morphology, as well as reducing body weight and ameliorates insulin sensitivity [14].

The activity of EGCG on pathways such as TLR4/NFkb and PI3K/AKT/FoxO1 is essential for attenuating inflammation, endoplasmic reticulum stress, oxidative stress, and fibrosis in NAFLD [10]. In addition, green tea extract increases the expression of genes related to lipid oxidation, preventing the accumulation of liver fat through the activation of AMPK [11, 13]. Despite the evidence of the beneficial effects of the green tea and its compounds against NAFLD, the mechanism by which polyphenols exert their effects on the different pathways highlighted here has not been fully elucidated.

In the last decade, the discovery of epigenetic mechanisms of small noncoding RNAs known as microRNAs (miRNAs) has emerged as potential therapeutic targets for several diseases [15]. Increasing evidence suggests that NAFLD is related to alterations in miRNA expression [16–20]. Jin et al. reported for the first time the difference in the pattern of miRNA expression between NAFLD and normal liver, evidencing the possible contribution of miRNAs for NAFLD pathogenesis and its potential as therapeutic targets and diagnosis [21]. miR-122 is the major miRNA known to be differentially expressed during NAFLD, and it is currently suggested as a predictive marker of fibrosis [22]. The link between miRNA and NAFLD is in the ability of these small molecules to modulate cellular metabolism, especially lipid metabolism in hepatocytes. Recently, it has been observed that the increase of miR-34a is responsible for the decrease of PPAR-*α* expression and consequent steatosis development [23]. In addition, inhibition of hepatic miR-24 leads to an increase in the *Insig1* target, a lipogenesis inhibitor, preventing hepatic lipid accumulation [3]. These data highlight the potential use of miRNAs as biomarkers and druggable targets in the search for new preventive and therapeutic strategies.

Recent evidence has been shown that polyphenols can modulate the expression of a hundred different miRNAs, which most of them involved in the control of inflammation, apoptosis, lipid metabolism, and insulin sensitivity [24]. The epigallocatechin-3-gallate (EGCG), the main polyphenol present in green tea (GT), is able to modulate the expression of several miRNAs in hepatocytes [25, 26]. Some studies suggest that the prevention of NAFLD in polyphenol-treated animals is associated with miRNA modulation [27, 28]. Also, it has been demonstrated that the administration of plant-derived polyphenols prevents hepatic steatosis in association with changes in the expression of miR-103, miR-107, and miR-122 [27]. In this context, this study is aimed at evaluating the hepatoprotective role of GT in a high-fat diet (HFD) mouse model of NAFLD and at determining the involvement of miRNAs *in viv*o and *in vitro*.

## 2. Material and Methods

### 2.1. Animals and Green Tea Supplementation

Twelve-week-old male C57Bl/6 mice were obtained from the University of São Paulo (USP, Brazil) and were housed in the animal facility of the Cruzeiro do Sul University for a one-week period of acclimation. Mice were kept in a room with controlled temperature (24 ± 2°C), light/dark cycle 12 : 12, and were given water and balanced diet (NUVILAB-CR1, Nuvital Nutrientes LTDA, Brazil) *ad libitum*. This study has been approved by the Ethics Committee on Animal Experimentation of the Cruzeiro do Sul University (CEUA Cruzeiro do Sul, 135/2014) and all animals received humane care according to the criteria outlined in the *Guide for the Care and Use of Laboratory Animals*. A total of thirty mice were randomly divided into 3 groups: (i) control (Cont) fed with chow diet, (ii) high-fat diet (HFD) fed with hyperlipidemic diet, and (iii) high-fat diet supplemented with green tea (HFD+GT). The protocol was performed twice with fifteen animals each. The chow diet consisted of 32% protein, 55% carbohydrates, and 13% lipids (2.88 kcal/g). On the other hand, HFD (PragSoluções, São Paulo, Brazil) was composed of 20% protein, 36% carbohydrates, and 34% lipids (5.31 kcal/g). Animals were fed an HFD *ad libitum*, for 16 weeks. After the 4^th^ week, the animals were submitted to a daily (Monday through Friday) oral gavage with 500 mg/kg body weight (BW) of green tea aqueous extract (HFD+GT group) or vehicle (Cont and HFD) for over 12 weeks. The powdered green tea extract was commercially acquired from Tovani Benzaquen, São Paulo, SP, Brazil. The dose of GT was based on previous *in vivo* studies from our group [29, 30]. The human equivalent dose (HED) was determined following equation: HED (mg/kg = animal NOAEL mg/kg) × (weight animal (kg)/weight human (kg)) (^1–0.67^). The dose by the factor method applies an exponent for body surface area, which accounts for the difference in metabolic rate, to convert doses between animals and humans [31]. HED = 500 mg/kg × (0.022 kg/70 kg)^0.33^ = 34.9 mg/kg or 2.44 g/70 kg. Therefore, we believe that until a total of 2.44 g/day/70 kg could be used in humans to promote health beneficial effects. That dose of GT can easily be ingested as capsules as a nutraceutical supplement and is therefore of physiological relevance. GT extract was weighed daily and then solubilized in distilled water at 70°C. After GT was at room temperature, it was administered by gavage to mice prior to the feeding period (between 6 : 00–7 : 00 pm) in a final volume of 100 *μ*L. The GT extract and standards (EC, EGCG, EGC, ECG, catechin, quercetin, and caffeine) were analyzed in an analytical LC (Varian 210) system with a ternary solvent delivery system equipped with an autosampler, a photodiode array detector (PDA) monitored at *λ* = 200‐800 nm. The catechins, quercetin, and caffeine were identified in GT extract by comparing their retention time with those of standard solutions. As obtained by the HPLC analysis, polyphenol, catechin, and caffeine contents in the extract were 39%, 30%, and 0.4%, respectively [29].

After 16 weeks into the experimental protocol, animals were euthanized by decapitation (between 9 : 00 and 12 : 00 h). Blood samples were drawn into heparinized tubes and centrifuged for 10 min at 1200×g, and plasma was stored at −80°C, to the subsequent determination of triglycerides (TG), aspartate aminotransferase (AST), and alanine transaminase (ALT) as well as the determination of cytokines and adipokines. The Aspartate Aminotransferase Activity Assay Kit provides a simple and direct procedure for measuring AST activity. In this kit, the transfer of an amino group from aspartate to *α*-ketoglutarate results in the generation of glutamate, resulting in the production of a colorimetric (450 nm) product proportional to the AST enzymatic activity present. One unit of AST is the amount of enzyme that will generate 1.0 *μ*mol of glutamate per minute at pH 8.0 at 37°C. The ALT Activity Assay Kit provides a simple and direct procedure for measuring ALT activity. ALT activity is determined by a coupled enzyme assay, which results in a colorimetric (570 nm)/fluorometric (*λ*ex = 535/*λ*em = 587 nm) product, proportional to the pyruvate generated. One unit of ALT is defined as the amount of enzyme that generates 1.0 *μ*mol of pyruvate per minute at 37°C. The tests were performed following the manufacturer's instructions. The liver was collected and fixed in 4% phosphate-buffered paraformaldehyde or snap-frozen in liquid nitrogen with storage at −80°C. Different white adipose tissue (WAT) depots—retroperitoneal, subcutaneous, and epididymal—and brown adipose tissue (BAT) were removed and weighed to calculate the adiposity index. Adiposity index was calculated as the sum of all the fat pad depots per animal and expressed per g/body weight (BW).

### 2.2. Assessment of Insulin Sensitivity

Fasting glucose was measured after 14 weeks through the experimental protocol as previously described in Rocha et al. [29]. A week prior to euthanasia, insulin tolerance test (ITT), and the rate of glucose clearance per minute (kITT) were performed and calculated as described by our group [29].

### 2.3. Examination of Liver Histopathology

Liver tissue was fixed in 4% phosphate-buffered paraformaldehyde for 24 h. Fixed samples were dehydrated by sequentially increased ethanol concentrations, cleared in xylene, and then embedded in paraffin wax. The embedded samples were cut into 5 *μ*m sections and stained with hematoxylin and eosin (H&E). Histological assessment and scoring of steatosis, hepatocellular ballooning, and lobular inflammation were performed by a pathologist blinded to the study. NAFLD activity score (NAS) and fibrosis stage were established using the histological criteria outlined by Kleiner et al. [32]. The degree of macrovesicular steatosis was graded using the following 4-point scale, namely, grade 0, steatosis involving <5% of hepatocytes; grade 1, steatosis involving up to 33% of hepatocytes; grade 2, steatosis involving 33–66% of hepatocytes; and grade 3, steatosis involving >66% of hepatocytes. Lobular inflammation was also graded on a 4-point scale, namely, grade 0, no foci; grade 1, fewer than 2 foci per ×20 field; grade 2, 2 to 4 foci per ×20 field; and grade 3, more than 4 foci per ×20 field. Hepatocyte ballooning was graded on a 3-point scale: 0, none; 1, a few balloon cells; and 2, any/prominent balloon cells. For NAS, features of steatosis, lobular inflammation, and hepatocyte ballooning were combined with values from 0 to 8. The OCT-embedded (optimal cutting temperature) frozen livers were sectioned at 10 *μ*m with a cryostat to detect neutral lipids by Oil Red-O staining. Briefly, slides were fixed in 10% (*v*/*v*) formalin for 10 min, stained with Oil Red-O working solution (Sigma-Aldrich, St. Louis, MO, USA), and counterstained with hematoxylin.

### 2.4. Glycogen Analysis

Liver glycogen content was measured after extraction with KOH and precipitation with ethanol followed by determination of glucose through phenol-sulfuric hydrolysis [33]. Liver samples (60 mg) were placed in plastic tubes containing 1.0 mL of 6 N KOH and were incubated in a boiling water bath for 10 minutes until complete dissolution. 250 *μ*L of the homogenate was mixed to 3 mL of 95% ethanol and 100 *μ*L of 10% K_2_SO_4_. A cloudy white precipitate was formed and the supernatant was discharged after centrifuging (150g for three minutes). Afterward, 300 *μ*L of each sample was analyzed at a wavelength at 490 nm on a Tecan spectrophotometric reader (Salzburg, Austria). A standard curve of oyster glycogen (Sigma) was prepared for the final quantification, expressed in *μ*mol/60 mg of hepatic tissue.

### 2.5. Plasma Insulin and Cytokines/Chemokines and Adipokines

Plasmatic insulin, cytokines/chemokines (IL-1*β*, IL-6, TNF-*α*), and adiponectin levels were measured using Multiplex MAP magnetic bead-based multianalyte panels (Mouse Cytokine/Chemokine Panel I (Cat. No. MCYTOMAG-70K), Mouse Adipokine (Cat. No. MADKMAG-71K), and Mouse Adiponectin Single Plex (Cat. No. MADPNMAG-70K-01)) (Millipore, Billerica, MA, USA) according to the manufacturer's guidelines.

### 2.6. MicroRNA Expression Analysis

Total RNA was extracted using miRVANA miRNA isolation kit (Ambion, Foster City, USA), according to the manufacturer's instructions. Total RNA (200 ng/*μ*L) was reverse-transcribed using miScriptII RT kit (Qiagen®, USA) according to the manufacturer's instructions. After reverse transcription, the expression of miR-34a and miR-194 was evaluated by qRT-PCR (Table 1). The results were normalized to U6. The *Δ*Ct method (2−*ΔΔ*Ct) was used to calculate relative changes in miRNA expression.

### 2.7. HepG2 Cell Treatment

Human hepatoma cells were a gift from Dr. Bruno Cogliati (HepG2; University of São Paulo). Cells were maintained at 37°C in 5% carbon dioxide in high-glucose (11.1 mmol/L) Dulbecco's modified Eagle's medium (DMEM) with 10% FBS and 10,000 U/mL penicillin-streptomycin. The HepG2 cell line at 70% confluent was used to validate the expression of target genes of miR-34a and miR-194. Cells were transfected with miRNA mimics or inhibitors using Lipofectamine 3000 (Invitrogen) according to the manufacturer's instructions. For inhibition of miR-34a and miR-194, anti-hsa-miR-34a and anti-hsa-miR-194 were used at final concentration of 40 nM. For the overexpression of pre-hsa-miR-34a and pre-hsa-miR-194 (Ambion), they were also used at 40 nM. Cells were also treated with GT extract (0.19%) in the presence of miR-34a and anti-miR194. After 24 h of transfection, the cells were collected and stored at -80°C for further RNA extraction and qRT-PCR analysis of target genes (Table 1).

In another set of experiments, the HepG2 cells at 90% confluent were treated for 24 h with TNF-*α* (10 ng/mL) to evaluate if this cytokine can modulate the expression of miR-34a and miR-194. Moreover, cells were treated with 2 *μ*M of a mix of catechins (epigallocatechin-3-gallate 1 *μ*M; epicatechin gallate 300 nM; epigallocatechin 500 nM and epicatechin 200 nM) or with GT extract (0.19%) to prevent the TNF-*α* effect. After 24 h, cells were collected and stored at -80°C for further miRNA expression analysis (Table 1). The dose of catechins *in vitro* and GT extract used was previously obtained in our laboratory as an effective dose to reduce the triglyceride content in HepG2 cells (data not shown).

### 2.8. qRT-PCR Analysis

Total RNA from the liver and HepG2-treated cells was extracted as previously described [29]. Total RNA was quantified and its integrity confirmed using agarose gel electrophoresis. Total RNA (2 *μ*g) was used to synthetize cDNA using random primers and 200 U SuperScript II RNase H reverse transcriptase at 42°C for 50 min, following the manufacturer's instructions.

The qRT-PCR was carried out using 30 ng of cDNA and 5× HOT FIREPol EvaGreen® qPCR Mix Plus (Solis Biodyne), and gene expression was assessed in an Agilent AriaMx Real-Time PCR under the following conditions: 50°C for 2 min, 95°C for 10 min, and 40 cycles of 95°C for 15 s, 60°C for 20 s, and 72°C for 30 s. Dissociation protocols were used to test the effectiveness of the primers in amplifying the genes specifically. In our experiments, the results were normalized by constitutive gene control (18S), determined in accordance with the pattern of tissue expression in each tissue analyzed. To identify predicted or validated targets mRNAs for the selected miRNAs (miR-34a and miR-194), the miRWalk and DIANA-miRPath databases were used. The *Δ*Ct method (2−*ΔΔ*Ct) was used to calculate relative changes in mRNA/miRNA expression.

### 2.9. Statistical Analysis

Our results are given as mean ± SEM. The variance of the data was verified by the Levene test. The interaction (diet, D × green tea, GT) was evaluated through a factorial two-way ANOVA using Tukey as posttest (*P* < 0.05). The main effect (diet, D, and/or green tea treatment, GT) was assessed by a factorial two-way ANOVA (*P* < 0.05) when the interaction was not statistically significant. Student's *t*-test (*P* < 0.05) was used for in vitro cell analysis. We used SPSS/Windows version 22 statistical package (SPSS Inc., Chicago, IL, USA) and GraphPad Prism statistics software package version 5.0 for Windows (GraphPad Software, San Diego, CA, USA).

## 3. Results

### 3.1. Green Tea Prevents Obesity Attenuating Inflammation in HFD-Induced NAFLD

**Figure 1:**
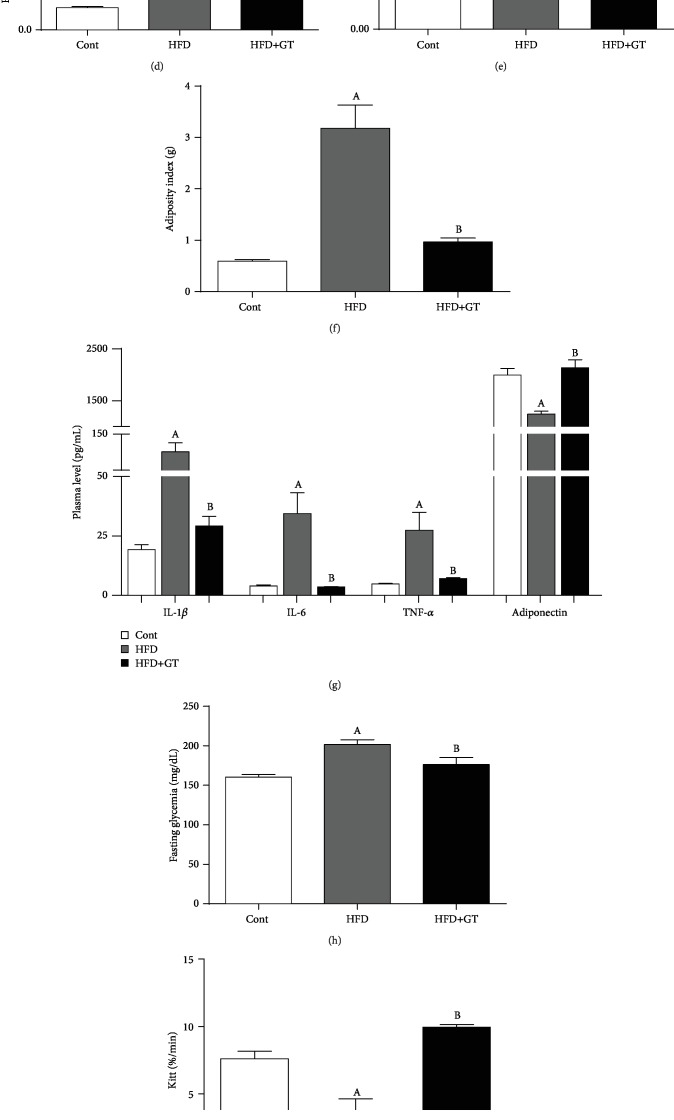
Green tea prevents obesity attenuating inflammation in HFD-fed mice with NAFLD. (a) Body weight, (b) body weight gain, (c) subcutaneous fatty depots, (d) epididymal, (e) brown adipose tissue, (f) adiposity index, (g) plasma levels of cytokines and adiponectin, (h) fasting glycemia, (i) calculation of the glucose decay constant in the bloodstream (kITT), (j) plasma insulin concentration, (k) HOMA index, (l) hepatic gene expression of *Ir*, *Irs2*, and *Pi3k*, (m) and hepatic glycogen content. The ITT assessment was performed at 0, 5, 15, 30, 60, and 90 minutes after intraperitoneal administration of insulin. Results are presented as mean ± SEM of at least 06 animals per group. ANOVA one- and two-way and Student's *t*-test were used for all the statistical analysis. When the difference was statistically significant, the following overwritten letters were used: ^a^Compared to the Cont group. ^b^Compared to the HFD group.

During the 16 experimental weeks, the body weight of the mice was evaluated. GT-supplemented mice showed attenuated body weight gain, decreased fat pad depots, resulting in a decreased adiposity index when compared to the HFD group (Figures 1(a)–1(f)). GT exhibited significant anti-inflammatory activity through the reduction of plasmatic inflammatory cytokine levels (IL-1*β*, IL-6, and TNF-*α*), also increasing plasmatic adiponectin level (Figure 1(g)) compared with the HFD group. Mice supplemented with GT exhibited lower fasting glycemia, plasma insulin level, and higher kITT, promoting lower HOMA index. Besides that, the liver of HFD+GT mice presented higher expression of insulin signaling genes such as *Irs2* and *Pi3k*, and higher hepatic glycogen depot (Figures 1(h)–1(m)). Altogether, these data indicate that GT supplementation protects against systemic insulin resistance (IR) improving hepatic insulin sensitivity.

### 3.2. Green Tea Has Hepatoprotective Effects in HFD-Induced NAFLD

**Figure 2:**
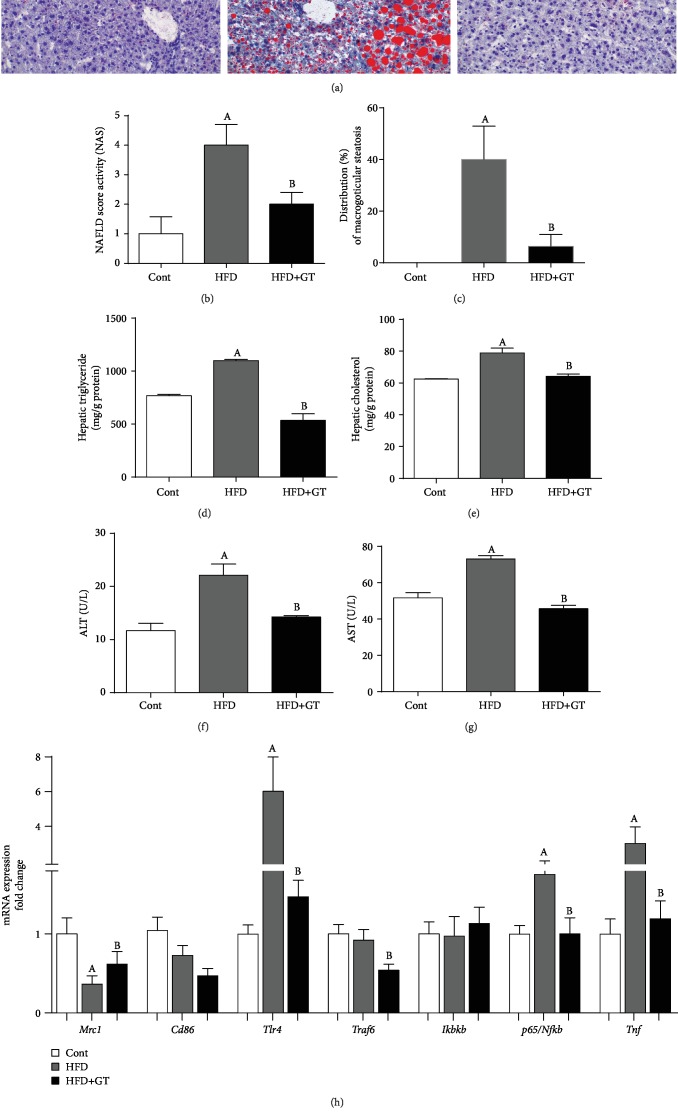
Green tea has hepatoprotective effects in HFD-induced NAFLD. (a) Liver histological analysis, (b) activity score of NAFLD, (c) distribution of macrovesicular steatosis, (d, e) hepatic triglyceride content and cholesterol, (f, g) ALT and AST plasma activity, and (h) analysis of expression of the genes involved in the inflammatory process, such as *Mcr1*, *Cd86*, *Tlr*, *Traf6*, *Ikbkb*, *Nfkb*, and *Tnf*. Results are presented as mean ± SEM of at least 06 animals per group. ANOVA one- and two-way and Student's *t*-test were used for all the statistical analysis. When the difference was statistically significant, the following overwritten letters were used: ^a^Compared to the Cont group. ^b^Compared to the HFD group.

To characterize our NAFLD model, we analyzed the hepatic lipid droplet accumulation through histological images using Oil Red-O staining and biochemical analysis of liver tissue. GT supplementation was effective in reducing lipid accumulation in the liver of HFD-fed mice (Figure 2(a)). GT-supplemented mice had lower activity score of NAFLD when compared to mice only fed with HFD (Figure 2(b)). Supplementation with GT reversed the changes in lipid profile induced by HFD in the liver reducing the macrovesicular steatosis, TG, and cholesterol content (Figures 2(c)–2(e)). We also evaluated the biochemical changes that contribute to the NAFLD characterization and we observed that the supplementation with GT minimizes liver damage induced by HFD (Figures 2(f) and 2(g)). To determine the inflammatory, liver profile evaluated the gene expression of some classical markers of inflammation. GT-supplemented mice showed higher expression of *Mrc1*, an anti-inflammatory macrophage (M2) marker, and lower expression of inflammatory genes *Tlr* (TLR4), *Traf6*, *Nfkb* (NF-*κ*B), and *Tnf* (TNF-*α*) when compared to the HFD group (Figure 2(h)).

### 3.3. Prevention of Steatosis by Green Tea Extract Is Mediated by Modulating of Lipid and Cholesterol Metabolic Genes

**Figure 3:**
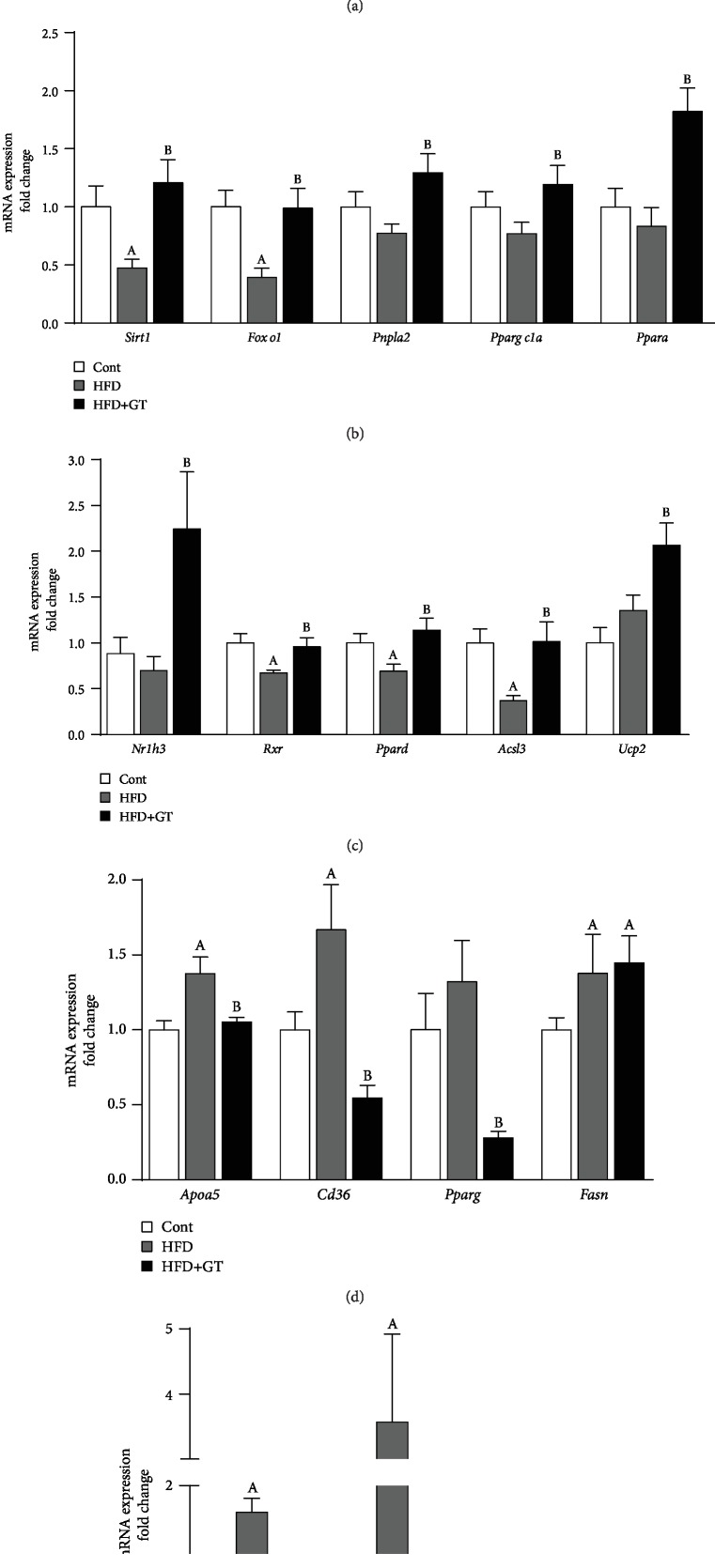
Prevention of steatosis by green tea extract is mediated by modulating of lipid and cholesterol metabolic genes. (a–c) Analysis of expression of genes involved in catabolism *Adipor1*, *Adipor2*, *Fgf21r*, *Prkacb*, *Prkaa2*, *Sirt1*, *Foxo1*, *Pnpla2*, *Ppargc1a*, *Ppara*, *Nr1h3*, *Rxrb*, *Ppard*, and *Acsl3*. (d) Genes associated with lipid accumulation (*Apoa5*, *Cd36*, *Pparg*, *Fasn*). (e) Genes associated with cholesterol biosynthesis (*Hmgcs2*, *Hmgcr*, *Insig2*). Results are presented as mean ± SEM of at least 06 animals per group. ANOVA one- and two-way and Student's *t*-test were used for all the statistical analysis. When the difference was statistically significant, the following overwritten letters were used: ^a^Compared to the Cont group. ^b^Compared to the HFD group.

To understand the metabolic changes that accompanied the phenotype of NAFLD, we evaluated the expression of key genes involved in the lipid and cholesterol hepatic metabolism. In general, the supplementation with GT increased lipid catabolism genes increasing the expression of *Adipor1/Adipor2* (adiponectin receptors) and *Fgf21r—*an important receptor that mediates lipid oxidation in the liver (Figure 3(a)). GT supplementation induces genes related to lipolysis and *β*-oxidation pathway, such as *Foxo1*, ATGL (*Pnpla2*), *β* subunit of PKA (*Prkacb*), *Sirt1*, *Rxrb* and *Acsl3*, AMPK alpha subunit (*Prkaa2*), PGC1-*α* (*Ppargc1a*), PPAR-*α*, LXR*α* (*Nr1h3*), and *Ucp2* when compared to the HFD group (Figures 3(a)–3(c)). Consistent with the histological analysis, we observed an increase in the expression of genes involved in TG accumulation, fatty acid (FA) uptake, and syntheses such as *Apoa5*, *CD36*, and *Fasn* in HFD-fed mice, while GT supplementation reverted *ApoA5* and *Cd36* expression, decreasing the transcription factor PPAR-*γ* (*Pparg*) (Figure 3(d)).

Regarding the cholesterol metabolism, we observed that GT supplementation reduces *Hmgcs2* (HMG-CoA synthase) and *Hmgcr* (HMG-CoA reductase) expression, key genes of cholesterol biosynthesis, while there is an increase in *Insig2* expression, responsible for HMG-CoA reductase cleavage and inactivation when compared to HFD group (Figure 3(e)). These data suggest that GT inhibits cholesterol biosynthesis, thereby decreasing the hepatic lipid content (Figure 2(e)).

### 3.4. Green Tea Modulates the Expression of miR-34a and miR-194 in Murine HFD-Induced NAFLD

To evaluate the involvement of miRNAs in the mechanism of action of GT, we evaluate the expression of miRNA 34a that is classically known to be involved in the regulation of lipid metabolism and is highly expressed in liver diseases. Also, miR-194 is poorly studied but it is highly expressed in the liver and has been implicated as a modulator of the inflammatory pathway [34].

**Figure 4:**
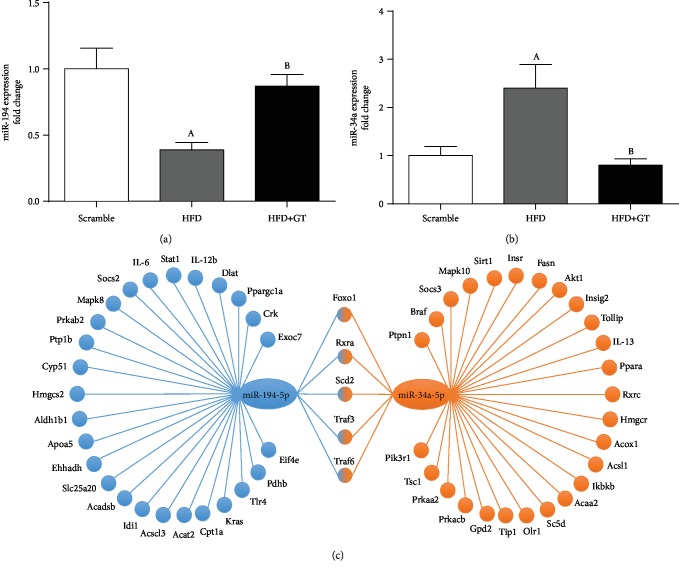
Green tea modulates the expression of miR-34a and miR-194 in murine HFD-induced NAFLD. Relative expression of miR-194 (a) and miR-34a (b). Results are presented as mean ± SEM of at least 06 animals. ANOVA one-way and two-way were used for the statistical analysis. When the difference was statistically significant, the following overwritten letters were used: ^a^Compared to the Scramble group. ^b^Compared to the HFD group. (c) Predicted and validated targets of the miRNAs: miR34a and miR-194. To select the targets, we used the miRWalk 2.0 database, based on the KEGG signaling pathway. Targets coregulated by different miRNAs or important members of the inflammation, metabolism, and IR were selected. Only targets present in 3 or more algorithmic bases were considered valid targets. We select predicted targets from prediction algorithms and validated targets (indicated by ∗) according to the literature. The genes presented at the intersection of the figure are target genes of both miR-34a and miR-194.

GT supplementation was effective in reversing miR-34a expression induced by HFD. Inversely, miR-194 was shown to be decreased in the NAFLD, whereas supplementation with green tea increased its expression (Figures 4(a) and 4(b)). After GT treatment, a positive correlation (*P* < 0.05) between miR-34a expression with mRNAs levels of *adipor2* (*r*^2^ = 0.66), *Prkacb* (*r*^2^ = 0.62), and *Hmgcs2* (*r*^2^ = 0.73) was found.

To identify predicted or validated targets mRNAs for the selected miRNAs (miR-34a and miR-194), the miRWalk and DIANA-miRPath databases were used. Initially, target genes involved in processes related to NAFLD pathogenesis such as glucose and IR, metabolism, inflammation, and lipid metabolism were selected. From this search, there were identified some targets that had their expression modulated by NAFLD and/or GT in an inverse manner to their respective miRNA, the selected genes were *Sirt1*, *Pparα*, and *Insig2* for miR-34a; *Apoa5* and *Hmgcs2* for miR-194 (Figure 4(c)). These targets were then selected for validation in HepG2 cells transfected with miR-34a or miR-194 mimetics and inhibitors, in order to outline the possible mechanism by which the modulation of these miRNAs is related with the development and prevention of NAFLD by HFD and GT, respectively.

### 3.5. Validation of Target mRNAs Regulated by miR-34a and miR-194 in HepG2 Cells

**Figure 5:**
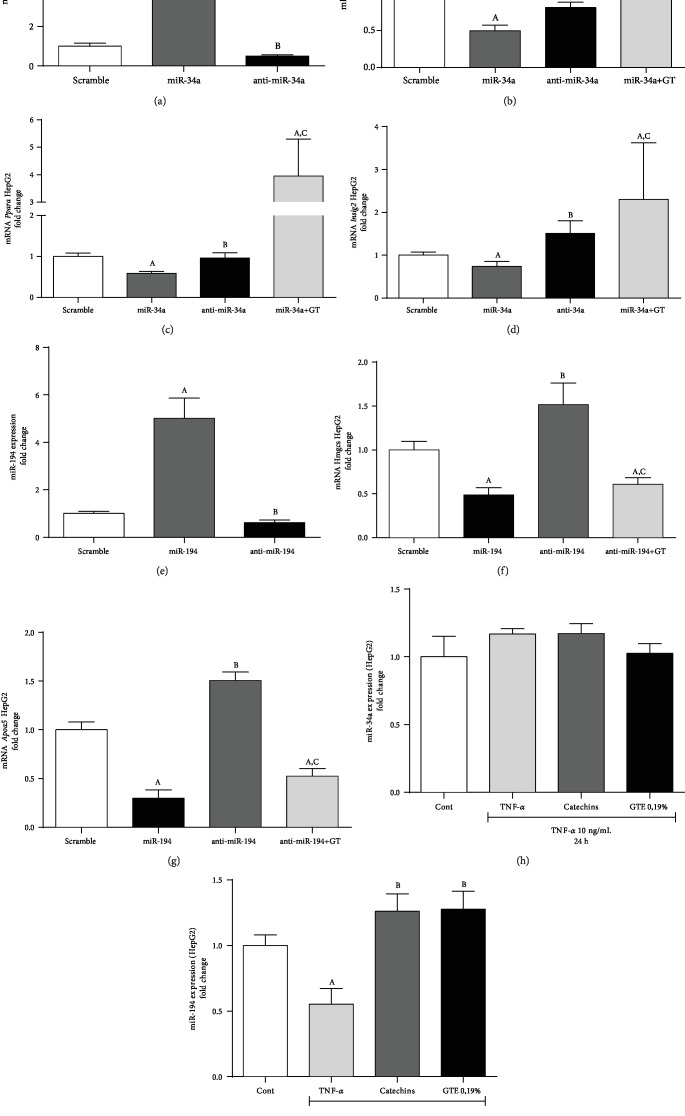
Validation of target mRNAs regulated by miR-34a and miR-194 in HepG2 cells. (a) miR-34a expression, (b) Sirt1, (c) *Ppara*, (d) *Insig2*, (e) miR-194 expression, (f) *Hmgcs2*, (g) *Apoa5*, (h) 34a expression, and (i) miR-194 expression. The results are presented as mean ± SEM of at least 05 in vitro transfection experiments. Student's *t*-test was used for the statistical analysis. When the difference was statistically significant, the following superscript letters were used: ^a^Compared to the Scramble group. ^b^Compared to the miR-34a, miR-194, or TNF-*α* group. ^c^Compared with respective group without GT.

We initially confirmed that transfection of anti-miR-34a and miR-34a mimic was effective in our cell system (Figure 5(a)). Consistent with our outcomes found *in vivo* in the HFD-fed mice, cells transfected with miR-34a mimic showed decreased *Sirt1*, *Insig2*, and *Ppara* expression. In addition, anti-miR-34a transfection leads to increased *Sirt1*, *Ppara*, and *Insig2* expression, similar to the results of mice in the HFD+GT group (Figures 5(b)–5(d)). Cells transfected with miR-34a and treated with GT have presented increased mRNA levels of *Sirt1*, *Ppara*, and *Insig2.* Taken together, these results suggest that the gene regulation of *Sirt1*, *Ppara*, and *Insig2* occurs through the modulation of miR-34a promoted by GT.

Transfection of HepG2 cells with miR-194 mimic and inhibitor was also effective (Figure 5(e)). We observed that anti-miR-194 transfection increased *Hmgcs2* expression when compared to the group transfected with miR-194 mimic, corroborating with the result found *in vivo* (HFD group). Similar to the GT-supplemented group *in vivo*, miR-194 mimic transfection decreased *Hmgcs2* expression. *Apoa5* expression was lower in cells transfected with miR-194 mimic as compared to the anti-miR-194 treated cells as observed in GT-supplemented mice compared to HFD mice (Figures 5(f) and 5(g)). GT treatment of cells transfected with anti-miR-194 has decreased both *Hmgcs2* and *Apoa5* mRNA expression. These results evidenced that *Hmgcs2* and *Apoa5* gene regulation occurs through miR-194 modulation. We found a positive correlation (*P* < 0.05) between miR-194 expression (*P* < 0.05) and mRNA levels of insig2 (*r*^2^ = 0.77), Foxo1 (*r*^2^ = 0.63), and adipor2 (*r* = 0.75) in HFD-fed mice and for insR (*r*^2^ = 0.56) in the HFD+GT group.

To understand the mechanism by which miR-34a and miR-194 are modulated in NAFLD condition, we evaluated their expression in HepG2 cells exposed to TNF-*α*, which was used to mimic the *in vivo* inflammation observed during NAFLD. The expression of miR-34a was independent of TNF-*α* exposure (Figure 5(h)), whereas the expression of miR-194 was directly affected by adding TNF-*α* in the cell culture. Interestingly, cells incubated with TNF-*α* and treated with a mix of catechins or the GT extract restored the expression of miR-194 (Figure 5(i)). These results support the idea that TNF-*α* regulates miR-194 expression and the modulation of GT occurs through its anti-inflammatory activity.

## 4. Discussion

In the present study, we demonstrate that the GT supplementation protects against obesity and NAFLD development in HFD-fed mice by altering lipid metabolism, increasing genes involved in TG catabolism and fatty acid oxidation, and also decreasing lipid uptake and accumulation as well as cholesterol synthesis. We have some evidence that the underlying mechanism by which the GT alters hepatic metabolism could involve epigenetic regulation of miR-34a and miR-194 that control the expression of target genes such as *Sirt1*/*Ppara*/*Insig2* and *Hmgcs2*/*Apoa5*, respectively. The mechanism by which miR-34a modulation occurs during NAFLD remains unknown, although we have demonstrated that miR-194 expression is downregulated by TNF-*α* and it was restored by GT.

Our experimental model of HFD was able to induce NAFLD in mice. In contrast, the livers from mice supplemented with green tea extract were protected against the lipid accumulation and inflammation. The GT extract dose used in this study was based in a systematic review of the literature published up to 2013 [35] that concluded, based on an analysis of the reports from 34 randomized clinical trials, that liver-related adverse events were rare after GT supplementation (seven cases in 1405 human subjects that had received dried GT extracts versus one case in 1200 controls) and that liver-related adverse effects were generally mild. Mice fed with HFD showed higher expression of hepatic genes involved in lipid accumulation pathways (*Apoa5*, *Cd36*, and *Fas*). Green tea supplementation decreased genes involved in lipid accumulation via such as *Cd36* and *Apoa5* and increased the expression of key genes involved in FA catabolism such as *Pnpla2*, *Fgf21r*, *Adipor1*, *Adipor2*, *Prkacb*, an *α*-catalytic subunit of *Prkaa2*, *Sirt1*, *Pgc1a*, *Nr1h3*, *Rxrb*, *Ppard*, *Ppara*, *Acsl3*, and *Ucp.* Taken together, these results indicate that green tea is responsible for lower uptake and lipid deposition, enhancing lipid catabolism and protecting the liver from steatosis.

A wide range of dietary factors, including micronutrients and bioactive compounds such as polyphenols, can modify the expression of miRNAs; additionally, these small regulators have significant contribution in pathways related to the development of NAFLD [24, 36]. Recently, miR-34a has been reported as a miRNA specifically modulated in liver diseases. Circulating levels of miR-34a are high in NAFLD patients as well as its hepatic expression in animal models of steatosis [23, 37]. Similarly, our NAFLD model showed increased hepatic expression of miR-34a, whereas the GT supplementation mitigates that induction. miR-34a expression in HFD-fed mice was positively correlated with liver cholesterol level (*r*^2^ = 0.98), TNF-*α* (*r*^2^ = 0.69), and steatosis (*r*^2^ = 0.66). Important lipid-related genes that were modulated in our *in vivo* study are a target of miR-34a. The genes *Sirt1*, *Ppara*, and *Insig2* had their expression inversely proportional to miR-34a expression *in vivo* and *in vitro* and GT treatment *in vitro* abolished miR-34a inhibitory effects on *Sirt1*, *Ppara*, and *Insig2* suggesting a direct effect of GT on miR-34a. Yamakuchi et al. have demonstrated that the *Sirt1* is a direct target of miR-34a [38]. In agreement with that data, our mice with NAFLD showed a decrease in the expression of Sirt1 while supplementation with green tea has reverted Sirt1 expression. Furthermore, Ding et al. [23] reported that inhibition of miRNA-34a improves hepatic steatosis of mice by increasing the expression of *Ppara* and *Sirt1*, enhancing phosphorylation of AMPK*α* and, consequently, increasing lipid oxidation. Our results suggest that green tea protects against NAFLD by enhancing the SIRT1/AMPK/PPAR-*α* signaling axis leading to increased lipid oxidation, with miR-34a mediating those effects corroborating with phenotype o liver tissue observed in histological images.

MiR-194 is known by downregulating the TLR4 signaling pathway through suppression of TRAF6 [39]. Although poorly studied, miR-194 presented an interesting modulation being negatively regulated during NAFLD and restored after green tea supplementation. Target genes involved in lipid and cholesterol metabolism, such as *Apoa5* and *Hmgcs2*, had their expression inversely proportional to miR-194 expression.

Camporez et al. [40] reported that ApoA5 knockout mice fed with HFD showed improvement in systemic insulin sensitivity and reduced liver steatosis. Corroborating with that the expression of miR-94 was decreased in the liver of HFD-fed mice beside an increase of *Apoa5* that could, along with other factors, be responsible for the impairment of insulin sensitivity and liver steatosis observed in our study. Positive modulation of miR-194 expression promoted by green tea could be responsible for the decrease of *Apoa5* gene expression and improved insulin sensitivity observed, as well as prevention of hepatic fatty accumulation. Our *in vitro* outcomes validate the modulation of all selected target genes with their respective miRNAs, evidencing that the protective effects of GT on the liver are mediated by direct regulation of miR-34a and miR-194.

It is well established that NAFLD development and evolution are influenced by cytokines and adipokines from adipose tissue, establishing a communication between adipose and hepatic tissues [41]. This crosstalk mediates several events and metabolic alterations [42]. TNF-*α* has been extensively investigated in this context, and several studies have revealed the regulation of different miRNAs (e.g., miR-142, miR-155, miR-335) by this cytokine [43, 44]. Recently, our group demonstrated that GT prevents obesity and improves adipose tissue metabolism through the miR-335 regulation mediated by TNF-*α* repression [45].

**Figure 6:**
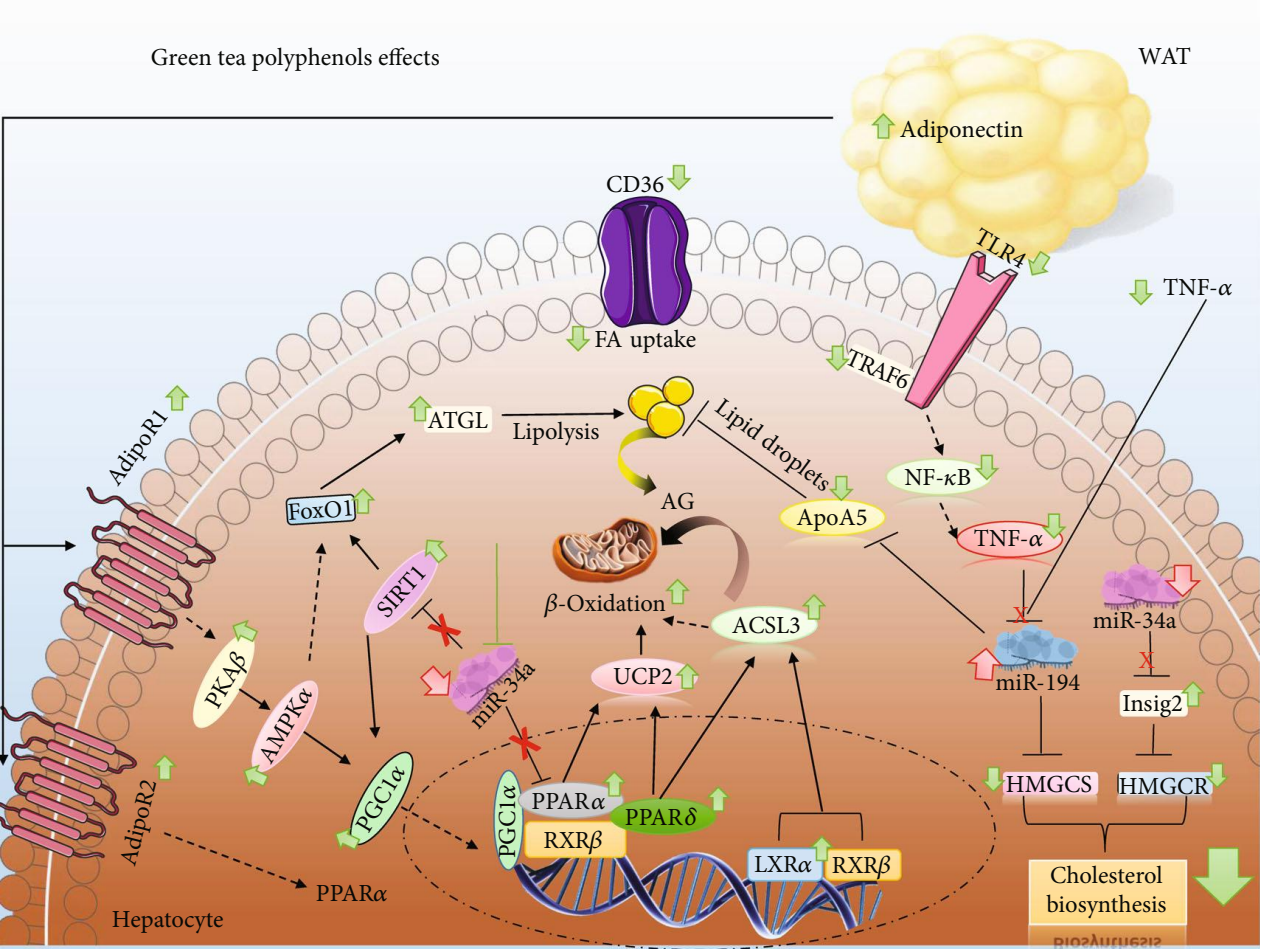
The proposed mechanism by which green tea acts against NAFLD. The reduction of hepatic lipid accumulation mediated by green tea during NAFLD occurs by a positive and negative regulation of FA oxidation and cholesterol synthesis, respectively. The decrease in miR-34a expression leads to the increase of its targets SIRT1 and PPAR-*α*, key genes for *β*-oxidation. High plasma levels of plasma adiponectin combined with the increase of its AdipoR1 and AdipoR2 receptors in the liver contribute to the activation of the AMPK pathway through the increase of PKA*β* and AMPK*α*. Both AMPK and SIRT1 are PGC1*α* activators. Increasing PGC1*α* together with increased RXR*β* results in increased PPAR-*α* and *δ* expression leading to the increase of UCP2 and ACSL3 that coordinate FA catabolism. To increase FA availability during this catabolism, an induction of FoxO1 is medicated by SIRT1/AMPK that results in the increase of ATGL, promoting a higher rate of lipolysis. To avoid ectopic fat accumulation, there is also a lower expression of CD36, avoiding the FA uptake from diet or adipose tissue. The decrease in the inflammatory axis TRL4/TRAF6/NFkB results in the decrease of TNF-*α*, a negative regulator of miR-194. Thus, hepatic and plasma TNF-*α* decrease mediates the increase of miR-194 which inhibits ApoA5 expression decreasing the lipid droplet formation, in addition to inhibiting HMGCS. Low expression of miR-34a increases Insig2 expression promoting the degradation of HMGCR. The decrease in HMGCS and HMGCR results in lower cholesterol biosynthesis and accumulation in the liver.

In view of the notoriety of TNF-*α* in regulating miRNA expression accompanied of increased plasma level and hepatic expression of TNF-*α* found in our study, we decided to evaluate the *in vitro* regulation of miR-34a and miR-194 in cells exposed to TNF-*α* and treated with catechins or GT extract. TNF-*α* was able to downregulate the miR-194 expression similar to observed in our HFD-induced NAFLD mouse model. HepG2 cells treated with catechins or GT extract were able to restore miR-194 expression to basal levels, similarl to the profile found *in vivo* following GT supplementation. These data suggest that TNF-*α* regulates miR-194 expression and the restoration of miR-194 by GT occurs through its anti-inflammatory activity. The modulation of miR-194 via TNF-*α* has evidenced a crosstalk between adipose tissue and liver mediated by adipocytokines. Since GT also increases adiponectin plasma levels as well as their hepatic receptors AdipoR1/R2 that are responsible for promoting beneficial metabolic actions, we are also suggesting the involvement of adiponectin as a mediator of the GT mechanism of action as proposed and presented in Figure 6.

In summary, this study has provided new evidence that the protective effects of GT in the liver presenting NAFLD are at least in part, mediated by miR-34a and miR-194 modulation. This study showed for the first time important findings such as the modulation of *Insig2* by miR-34a, the involvement of miR-194 in lipid and hepatic cholesterol metabolism, and their regulation by TNF-*α*. However, further studies are needed to determine whether the modulation of miR-194 by GT and its catechins is mediated by an anti-inflammatory effect on TNF-*α* or by a direct effect on the expression of miRNA-194. Understanding the pathways shown herein reveals new targets for the prevention and therapeutic intervention for NAFLD and evidence of the involvement of miR-34a and miR-194 in the mechanism of action of green tea polyphenols.

## Figures and Tables

**Table 1:** Nucleotide sequences of primers.

Gene	Primer sense (5′ to 3′)	Primer anti-sense (5′ to 3′)
*miR-34a-5p*	TGGCAGTGTCTTAGCTGGTTG	
*miR-194-5p*	TGTAACAGCAACTCCATGTGGA	
Universal_MS2_R		CGAGGTCGACTTCCTAGATTTTT
*hsa-18s*	TGAGAAACGGCTACCACATC	TTACAGGGCCTCGAAAGAGT
*hsa-Apoa5*	TGGCTCTTCTTTCAGCGTTT	TTGCTCAAGGCTGTCTTTCA
*hsa-Hmgcs2*	TATAAGGGGCTGGAGGCTTT	CATGTTCCCATTGTGAGTGG
*hsa-Insig2*	GTCCAGTGTAATTGCGGTGTG	GAGTGACCACAGTTGCCAAG
*hsa-Ppara*	GCCTACAGGCTATCATTACGG	GTTGTGTGACATCCCGACAG
*hsa-Sirt1*	CCGGATTTGAAGAATGTTGG	AGCGCCATGGAAAATGTAAC
*mmu-18s*	CTCAACACGGGAAACCTCAC	CGCTCCACCAACTAAGAACG
*mmu-Acs13*	GAGGTCCAGCCATTGTTCAT	CAATGACACCTTTGGGGAAC
*mmu-Adipor1*	AGGCTGAGGAAGATCAAGCA	CGTTGTCTTTCAGCCAGTCA
*mmu-Adipor2*	GGAGTGTTCGTGGGCTTAGG	GCAGCTCCGGTGATATAGAGG
*mmu-Apoa5*	GAGTCGAGTGCTGCACCATA	TCGCCTTACGTGTGAGTTTG
*mmu-Cd36*	TGGAGCTGTTATTGGTGCAG	TGGGTTTTGCACATCAAAGA
*mmu-Cd86*	GACCGTTGTGTGTGTTCTGG	GATGAGCAGCATCACAAGGA
*mmu-Fasn*	TATCAAGGAGGCCCATTTTGC	TGTTTCCACTTCTAAACCATGCT
*mmu-Fgf21r*	CTGCTGGGGGTCTACCAAG	CTGCGCCTACCACTGTTCC
*mmu-Foxo1*	GTGAACACCATGCCTCACAC	ACTTGGGAGCTTCTCCTGGT
*mmu-Hmgcr*	CTCCTCTCCACAAAGCTTGC	CTGGTACTCCCATCCA
*mmu-Hmgcs2*	AGGACATCAACTCCCTGTGC	TCAGTGTTGCCTGAATCCTG
*mmu-Ikbkb*	AGCTGTCCTTACCCTGCTGA	AAATGACGTGCACAGACTGC
*mmu-Insig2*	AGTGTGGCCCATACATTTCC	GCTCGTGATCACATCTGGTG
*mmu-Ir*	GGATGTGACAGCCACCACAC	CTGGGGATTCTTGATTGCAT
*mmu-Irs2*	CTGCGTCCTCTCCCAAAGTG	GGGGTCATGGGCATGTAGC
*mmu-Mcr1*	AACAAGAATGGTGGGCAGTC	TTTGCAAAGTTGGGTTCTCC
*mmu-Nfkb*	GTAACAGCAGGACCCAAGGA	TCCGCCTTCTGCTTGTAGAT
*mmu-Nrlh3*	GGATAGGGTTGGAGTCAGCA	GCTTTGTGTCCCCACAGACACT
*mmu-Pi3k*	AAAAATGGCGACGACTTACG	TTGCACTGGATTTGCATGAT
*mmu-Pnpla2*	GAGTGCAGTGTCCTTCACCA	CAGTTCCACCTGCTCAGACA
*mmu-PPrara*	CGACCTGAAAGATTCGGAAA	CTCGGCCATACACAAGGTCT
*mmu-PPrard*	CGAGTTCTTGCGAAGTCTCC	TGTCCTGGGATGGCTTCACAAG
*mmu-Pparg*	TCAGCTCTGTGGACCTCTCC	ACCCTTGCATCCTTCACAAG
*mmu-Ppargc1a*	CCCTGCCATTGTTAAGACC	TGCTGCTGTTCCTGTTTTC
*mmu-Prkaa2*	GTGATCAGCACTCCGACAGA	TCTCTGGCTTCAGGTCCCTA
*mmu-Prkacb*	GGAGATCATCCTCAGCAAGG	GCAGAAGGTCCTTGAGATCG
*mmu-Rxrb*	AGTGTCCAAAATGCGTGACA	GCTGCTCAGGGTACTTCTGC
*mmu-Sirt1*	TCTCCTGTGGGATTCCTGAC	ACACAGAGACGGCTGGAACT
*mmu-Tlr*	CAGCAAAGTCCCTGATGACA	TGGCATGGACTAAGGATGTG
*mmu-Tnf*	CCACCACGCTCTTCTGTCTA	GATCTGAGTGTGAGGGTCTGG
*mmu-Traf6*	GCCCAGGCTGTTCATAATGT	AGCTCGCCCACGTACATACT
*mmu-Ucp2*	GGTCGGAGATACCAGAGCAC	TGTCATGAGGTTGGCTTTCA
*mmu-Xbp1s*	TTACGAGAGAAAACTCATGGGC	GGGTCCAACTTGTCCAGAATGC

## Data Availability

Data availability will be provided when requested.
